# Medical Schools

**Published:** 1850-08

**Authors:** 


### MEDICAL SCHOOLS.

The season of medical lectures is approaching, and we hope that students of medicine, throughout the country are preparing themselves for the lecture terms by a very vigorous prosecution of studies. Nothing can so much tend to exalt the profession as a universal aspiration of each student for the highest amount of knowledge that can be attained by industry and devotion, and wherever that is most accessible, there students should congregate.

Of our judgement of the merits of the Medical Department of the University of Louisville, the readers of this Journal are well advised. We are wedded to no school in such a way as to blind us as to its faults or deficiencies. Our connection with this Journal is as independent of any school of medicine as though there were none in this city, and we may speak of that school as our judgement may lead us to do. And in full view of this responsibility, we run no hazard in saying that in that important element of the success of a medical school, the Chair of Theory and Practice, we know of no one that offers such facilities for acquiring a full knowledge of the philosophy of medicine, as those presented by the Louisville Medical School.

Of our colleague in this Journal, who is far remote from Louisville at this time, and who can therefore, exert no influence upon this paragraph, we can speak in befitting terms. He has been engaged, since the close of last session, in a vigorous prosecution of microscopic physiology, and we entertain no kind of doubt, that he will deliver, during the coming winter, a course of lectures, at once creditable to himself, and to the institution, of which he is a distinguished member. This much we feel is due to him.

But we would earnestly impress upon students the vast importance of self-culture. No extrinsic advantages can ever make amends for deficiences in that important element of success. We pray them, therefore, to enter the classes, thoroughly prepared in everything that can be accomplished by thorough study. Let no one be content with merely rubbing through the green room, but let it be the object of each one to see how thoroughly he can understand everything connected with the important responsibilities he assumes in becoming a practitioner of medicine. He should feel that it is no ordinary matter to take charge of the health and prosperity of a community. B.

